# Galactosylated Albumin Nanoparticles of Simvastatin 

**Published:** 2015

**Authors:** Kumar Ganesh, Dhyani Archana, Kothiyal Preeti

**Affiliations:** *Division of Pharmaceutical Sciences, Sri Guru Ram Rai Institute of Technology and Sciences, Patel Nagar, Dehradun.*

**Keywords:** Hepatotoxicity, Galactose, Targeting, Asialoglycoprotein receptor

## Abstract

The present study was an attempt to develop galactosylated albumin nanoparticles of Simvastatin for treatment of hypercholesterolemia. By developing the galactosylated nanoparticulated delivery, the required action of the drug at the target site at the liver can be provided. The advantage of targeting helps to reduce the systemic side effects that may occur due to the distribution of the drug to the other organs and thus helps in maintaining the required concentration of drug at the desired site. The galacotsylated albumin nanoparticles were prepared for the selective delivery of a Simvastatin to the 3-hydroxy-3-methylglutaryl coenzyme A reductase (HMG-CoA reductase) the rate-limiting enzyme in the pathway of cholesterol biosynthesis that is particularly presents on hepatocytes. The asialoglycoprotein receptor (ASGP-R) which is particularly presents on mammalian hepatocytes can be utilize for active targeting by using its natural and synthetic ligands. By utilizing this receptors can provides a unique means for the development of liver-specific carriers, such as liposomes, recombinant lipoproteins, and polymers for drug or gene delivery to the liver, especially to hepatocytes. These receptors recognize the ligands with terminal galactose or N-acetylgalactosamine residues, and endocytose the ligands for an intracellular degradation process.

The albumin nanoparticles (NPs) were prepared by using desolvation method and efficiently conjugated with galactose. Various parameters such as particle size, zeta potential, percentage entrapment efficiency and drug loading efficiency, percentage yield, *in-vitro* drug release were determined. The size of nanoparticles (both plain and coated NPs) was 200 and 250 nm. The zeta potential of plain nanoparticles was -3.61 and that of galactose-coated nanoparticles was 64.1. The maximum drug content was in between 79.98% to 79.8 % respectively in plain, and galactose coated nanoparticles while the maximum entrapment efficiency was 70.10% and 71.03% in plain and coated nanoparticles. It was found that coating of nanoparticles increases the size of nanoparticles.

## Introduction

The liver is the primary organ for regulation of total body cholesterol homeostasis in mammalian systems. Hepatic coordination of cholesterol biosynthesis with assembly, secretion, and uptake of plasma lipoproteins depends in part on cellular mechanisms coupling the activities of the key enzymes of sterol synthesis with the receptors governing lipoprotein clearance (1). Thus an important target for pharmacological regulation of plasma low density lipoprotein cholesterol is liver 3-hydroxy-3-methylglutaryl coenzyme A reductase (HMG-CoA reductase) the rate limiting enzyme in the pathway of cholesterol biosynthesis.

The low solubility and stability of drug in physiological environment are the main problems in attaining the bioavailability. Several approaches are used in order to increase the solubility, stability, bioavailability, *etc.* aspects of drugs. Among them, polymeric nanoparticles, dendrimers, polymeric micelles and polymersomes appear as the most attractive and promising (2, 3).

Dyslipidemia, including hypercholesterolemia, hypertriglyceridemia, or their combination, is amajor factor for cardiovascular disease. Generally, dyslipidemia is characterized by increased fasting concentrations of total cholesterol (TC), triglycerides (TG), and low-density lipoprotein cholesterol (LDL-C), in conjunction with decreased concentrations of high-density lipoprotein cholesterol (HDL-C). At present, these lipid imbalances are most routinely treated with pharmacological therapy.

Simvastatin is a poorly soluble lipid-lowering agent, which is used for the treatment of primary hypercholesterolemia. When given orally, Simvastatin (a lactone) undergoes hydrolysis and is converted to the *β, δ*-dihydroxy acid form, a potent competitive inhibitor of 3-hydroxyglutaryl-CoA reductase the enzyme that catalyzes the rate-limiting step of cholesterol biosynthesis (4). Water solubility of Simvastatin is very low, approximately 30 μg/mL (5). It is practically insoluble in water and poorly absorbed from the gastrointestinal (GI) tract. Therefore, it is very important to introduce effective methods to enhance the solubility and dissolution rate of drug, substantially leading to its bioavailability. Improvement of the aqueous solubility in such a case is a valuable goal that leads to enhancing therapeutic efficacy (6). Here, the solubility of Simvastatin is increase by the addition of surfactants and reduction of particle size.

Albumin is an attractive macromolecular carrier and it is widely used to prepare nanospheres and nanocapsules, due to its various advantages like biodegradability, non-toxicity and non-immmunogenicity. Both Bovine Serum Albumin or BSA and Human Serum Albumin or HSA have been use. Albumin nanoparticles are biodegradable, easy to prepare in defined sizes, and carry reactive groups (thiol, amino, and carboxylic groups) on their surfaces that can be used for ligand binding and/or other surface modifications and nanoparticles offer the advantage that ligands can be easily attach by covalent linkage (7).

The three different methods for the preparation of nanoparticles are emulsification, desolvation, or coacervation. Most often serum albumin of different origin as well as gelatin was used as the starting material for the preparations. With respect to emulsion techniques applying human serum albumin (HSA), a complete and systematic study is concerning the influence of protein concentration, emulsification time and power, stirring rate, heat stabilization temperature, and the type of the non-aqueous phase (8). The disadvantage of the emulsion methods for particle preparation is the need for applying organic solvents, for the removal of both the oily residues of the preparation process and of surfactants required for emulsion stabilization. Therefore, as an alternative method for the preparation of nanoparticles a desolvation process derived from the coacervation method of microencapsulation was developed. In 1993, Lin *et al*. described the preparation of Human Serum Albumin nanoparticles of diameter around 100 nm using a surfactant-free pH-coacervation method (9). The particles were prepared by the drop wise addition of acetone to an aqueous Human Serum Albumin solution at pH values between 7 and 9, followed by glutaraldehyde cross-linking and purification by gel permeation chromatography. It was found that with increasing pH value of the Human Serum Albumin solution particle size was reduced, apparently due to an increased ionization of the HSA (isoelectric point pI = 5.3) which leads to repulsion of the Human Serum Albumin molecules and aggregates during particle formation. Human Serum Albumin nanoparticles were obtain in a size range between 90 and 250 nm, by adjusting the pH and by controlling the amount of added acetone.

## Experimental

*Materials*

Simvastatin was a gift sample from Ind. Swift Pharmaceutical *Ltd,* Chandigarh, sterile bovine serum albumin, sodium chloride, sodium lauryl sulfate, ethanol were obtained from Central Drug House *Ltd,* New Delhi. All the reagents and solvents used were of analytical grade satisfying Pharmacopeia standards.

**Table 1:** Composition of different Nanoparticle formulations

**Ingredients**	**Formulations**				
	**F1**	**F2**	**F3**	**F4**	**F5**
**Drug(mg)**	40	40	40	40	40
**BSA(mg)**	50	100	200	600	1000
**Ethanol (mL)**	8	8	8	8	8
**Glutaraldehyde(%)**	8	8	8	8	8
**Galactose(mg)**	20	20	20	20	20
**Sodium Lauryl Sulfate (%)**	0.5	0.5	0.5	0.5	0.5

*Preparation of* *bovine serum albumin* *nanoparticles*

Bovine Serum Albumin nanoparticles were prepared by a desolvation (10). In principle, between 50 and 1000 mg Bovine Serum Albumin was added in 2.0 mL of 10 mM NaCl solution, titrated to pH 8, the drug was also incorporated and addition of few mL of 0.5% Sodium Lauryl Sulfate concentration were transformed into nanoparticles by the continuous addition of 8.0 mL of the desolvating agent ethanol under stirring (500 rpm) at room temperature. After the desolvation process, 8% glutaraldehyde in water was added to induce particle crosslinking. The crosslinking process was performing under stirring of the suspension over a period of 24 h.

*Purification of BSA nanoparticles*

The resulting nanoparticles were purified by three cycles of differential centrifugation (20,000 rpm, 10 min) and redispersion of the pellet to the original volume in 10 mM NaCl at pH values of 8, respectively. Each redispersion step was performed in an ultrasonication bath over 5 min. The solvent was evaporated by rotary evaporator and the nanoparticles were stored at 2-8 ^ο ^C.

*Galactose coating of nanoparticles*

20 mg of galactose were added to 10 mg of BSA nanoparticles nanoparticles dispersed in 5 mL acidic PBS (pH 5.0), and the mixture was then stirred at room temperature over-night. The resulting nanoparticles were purified by three cycles of differential centrifugation (20,000 rpm, 10 min) and redispersion of the pellet to the original volume in water or 10 mM NaCl at pH values of 7 and 9, respectively. Each redispersion step was performed in an ultrasonication bath over 5 min. The solvent was evaporated by rotary evaporated and the nanoparticles were stored at 2-8 ^ο^C.

*Characterization of nanoparticles*

*Shape and Size*

The morphology of plain and galactose-coated nanoparticles was determined by Scanning electron microscopy.

*Zeta potential*

The Zeta Potential Analyzers determined the zeta potential and surface charge of nanoparticles. The zeta potential of nanoparticles is commonly use to characterize the surface charge property of nanoparticles.

*Drug content uniformity*

10 mg of nanoparticle was introduced in a 100 mL volumetric flask. The nanoparticles were dissolved in phosphate buffer pH 7.4 and make up the volume up to 100 mL. The above solution was analyze by UV spectrometer at 238 nm

*Entrapment efficiency*

10 mg of nanoparticle was took and introduced in a 100 mL volumetric flask. The nanoparticles were dissolved in phosphate buffer pH 7.4 and make up the volume up to 100 mL. The above solutions were analyzed by UV spectrometer at 238 nm. The entrapment efficiency of the prepared nanoparticles was calculated by the formula :

Entrapment efficiency%=Practical drug loading-Theoretical drug loading Theoretical drug loading×100

*Percentage yield*

It is calculate to know about the efficiency of any method, thus it helps in selection of appropriate method of production. Practical yield was calculated as the weight of nanoparticles recovered from each batch in relation to the sum of starting material.

It can be calculated using following formula:

$\mathrm{Percentage yield}=\frac{\mathrm{Practical yield}}{\mathrm{Theoretical yield}}\times 100$

*In-vitro drug release*

In -Modified Diffusion Apparatus carried *out-vitro* drug release. The apparatus consists of a beaker containing 50 mL of phosphate buffer pH 7.4 maintained at 37 ^ᵒ^C under mild agitation using a magnetic stirrer acts as receptor compartment. An open- ended tube acts as donor compartment and the egg membrane was tie into upper part of the donor compartment. The nanoparticles (plain and galactose coated) equivalent to 10 mg were placed in to the donor compartment over the membrane which was dipped in the receptor compartment consisting buffer. Then, the samples were taken at different time intervals from the receptor compartment and were analyze by UV spectrometer at 238 nm.

*Mathematical modeling*

Various conventional mathematical models (zero-order, first-order, Higuchi, Korsmeyer- Peppas) to determine the release mechanism from the designed nanoparticle formulations (10–12) treated the data obtained from *in-vitro* release studies^.^ Selection of a suitable release model was based on the values of R (correlation coefficient), k (release constant) and n (diffusion exponent) obtained from the curve fitting of release data. 

*Receptor ligand binding study*

After fasting overnight mice was killed by cervical dislocation, liver were excised, and homogenized with 0.1 M phosphate buffer pH 7.4. The homogenate was homogenized in 0.25 M sucrose containing EDTA (1 mM). The homogenate was centrifuge at 30,000 rpm for 10 min. The resulting supernatant was centrifuge at 10,000 rpm for 10 min. The supernatant was collected and suspended in the same buffer.

10 mg of nanoparticles were added into the supernatant containing hepatocytes and homogenized at a high speed (20,000 rpm) for 20 min. 5 mL of the solution was placed in donor compartment of Modified Diffusion Apparatus. Then, the samples were taken at definite time intervals from the receptor compartment and were analyze by UV spectrometer at 234 nm.

*Results and discussions*

Five formulations of Simvastatin were formulated using different drug polymer ratios. The formulation is subjected to evaluation parameters like particle size, percentage yield, entrapment efficiency, zeta potential, drug content uniformity, *in-vitro* drug release, ligand receptor binding study.

*Characterization of nanoparticles*

*Particle size*

The size of all batches of plain nanoparticles was found to be in the size of 200 nm and that of galactose coated nanoparticles was found to be in the size range of 250 nm.

**Figure 1:**
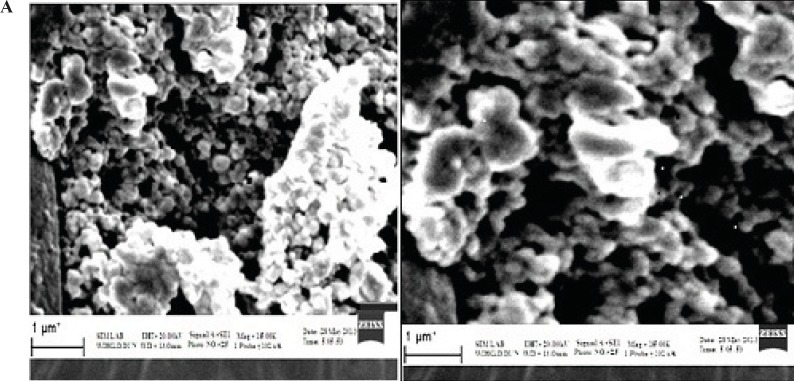
(A) Scanning electron microscopy (SEM) photomicrograph of Albumin-Nanoparticles; (B) SEM photomicrograph of Galactose coated Nanoparticles

The SEM photomicrographs of nanoparticles are shown in Figures 1 (A and B)*. *It was observed from these photomicrographs that all samples of particles were smooth, sub-spherical in shape and aggregated to form small clusters.

The larger particle size of galactosylated nanoparticles as compared to plain nanoparticles could be due to the anchoring of galactose molecule at the surface of nanoparticles and hence an increment in size of nanoparticles was observed.

*Zeta potential*

The Zeta Potential Analyzers determined the zeta potential and surface charge of nanoparticles. The zeta potential of nanoparticles is commonly use to characterize the surface charge property of nanoparticles.

**Figure 2:**
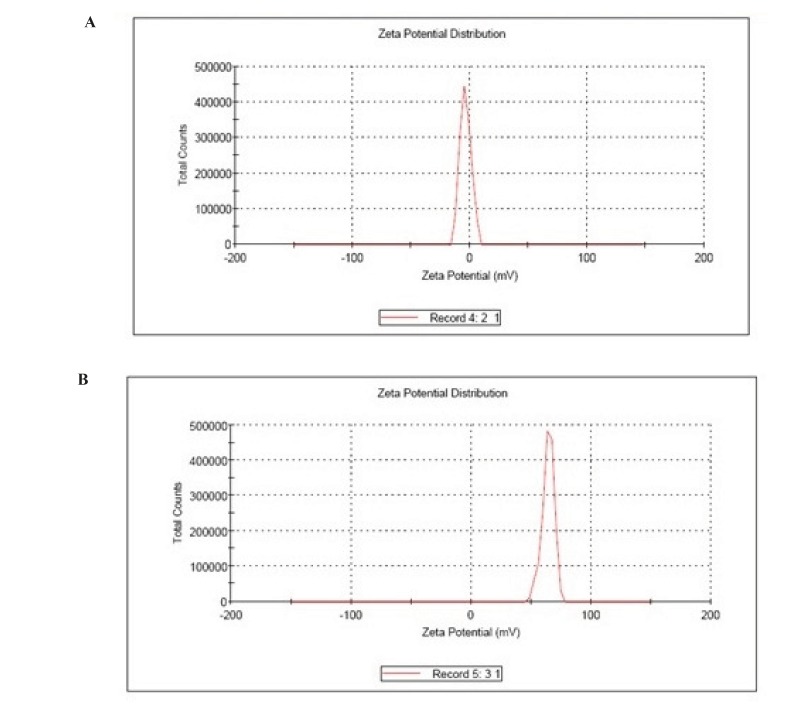
(A) Zeta potential of albumin nanoparticles, (B) Zeta Potential of galactose coated nanoparticles.

*Drug content uniformity*

The drug content of different formulations F1 to F5 was calculated and the content was found to be in range of 45.09 to 93.80% for plain nanoparticles and 46.8 to 95.98% for coated nanoparticles. The maximum drug content was found to be 93.8% for plain and 95.98 % for coated nanoparticles for the formulation F3. The nanoparticles exhibited an increase in drug content with an increased in polymer ratio, up to particular concentration. A decrease in drug content was observed after that point due to saturation capacity of polymer. The results are shown in Table 2. 

**Table 2:** Drug content of Simvastatin nanoparticles

**Formulation code**	**Drug content (%)**	
	**Plain nanoparticles**	**Coated nanoparticles**
**F1**	45.09	46.8
**F2**	55.12	57.81
**F3**	93.80	95.98
**F4**	82.09	84.09
**F5**	76.98	79.8

*Nanoparticulate yield*

The percentage yield of different formulations F1 to F5 was calculated and the yield was found to be in the range of 32.14 to 83.24% for plain nanoparticles and 28.75 to 79.8 % for coated nanoparticles. Percentage Yield of all batches is shown in Table 3. Maximum particle yield was found in F5 (83.24 % and 79.8% for plain and coated nanoparticles) where the concentration of albumin is highest while the nanoparticle yield is lowest in F1 (32.14% and 28.75% for plain and coated nanoparticles) where the concentration of albumin is low. 

The reduction in percentage yield after coating of nanoparticles might be occur due to the loss of nanopaticles during the coating process

**Table 3:** Percentage yield of Simvastatin nanoparticles

**Formulation code**	**Total amount of ingredients (mg)**		**Percentage yield (%)**	
	**Plain nanoparticles**	**Coated nanoparticles**	**Plain nanoparticles**	**Coated nanoparticles**
**F1**	90	110	32.14	28.75
**F2**	140	160	41.23	36.09
**F3**	240	260	55.74	51.29
**F4**	640	660	74.31	70.09
**F5**	1040	1060	83.24	79.8

*Entrapment efficiency*

The encapsulation efficiencies of all four formulations were given in the Table 4 and the entrapment efficiency were found to be in range of 32.19 to 90.91% for plain nanoparticles and 38.09% to 93.27 % for coated nanoparticles. The maximum entrapment efficiency was found to be 90.91% and 93.27 % for the formulation F3.

The relatively higher percent drug entrapment was obtained for coated nanoparticles as compared to the plain nanoparticles, which could be due to minimum repulsion between drug and polymer.

**Table 4:** Entrapment efficiency of Simvastatin nanoparticles

**Formulation code**	**Entrapment efficiency** ** (%)**	
	**Plain nanoparticles**	**Coated nanoparticles**
**F1**	32.19	38.09
**F2**	48.67	50.98
**F3**	90.91	93.27
**F4**	78.09	81.29
**F5**	70.10	71.03

*In-vitro release profile*

The comparative plot of the percent release profile of Simvastatin loaded BSA nanoparticles is shown in Figure 2(a and b). The key results obtained by evaluation of the percent release values are summarize in Table 5. As observed in Table 5, the overall highest release was observed in the formulation F1, which contained lowest amount of BSA (92.6 % after 10 h).

It was interpreted from the result that the formulation with the lowest polymer content showed the fastest release. In contrast, F5, which contained maximum BSA, showed minimum release (50.8 % after 10 h). Thus, it was found that the formulation with high polymer content showed the slowest release. It was also found that coating of nanoparticles with galactose also decreases the dug release F5(48.71% after 10 h).

**Figure 3:**
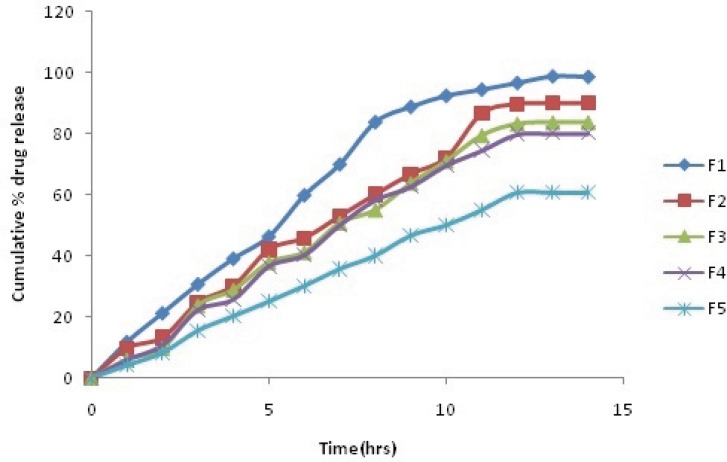
Zero order release Plot of Simvastatin plain nanoparticles

**Figure 4:**
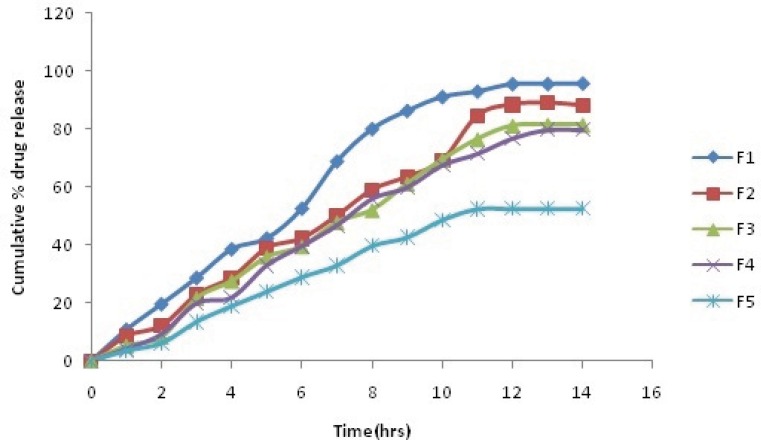
Zero order release Plot of Simvastatin galactose coated nanoparticles

**Table 5:** Cumulative % drug release of Plain and Galactose coated Nanoparticles

**Time(h)**	**Cumulative % drug release**									
	**F1**		**F2**		**F3**		**F4**		**F5**	
	**Plain**	**Coated**	**Plain**	**Coated**	**Plain**	**Coated**	**Plain**	**Coated**	**Plain**	**Coated**
1	11.84	10.84	9.76	8.54	6.06	5.21	5.92	4.12	4.16	3.45
2	21.31	19.65	13.26	12.12	9.78	8.25	10.76	9.23	8.29	6.08
3	30.78	28.73	24.7	22.87	23.79	21.65	22.26	19.87	15.59	13.52
4	39.19	38.63	30.18	28.72	28.9	27.56	25.78	21.87	20.29	18.9
5	46.42	42.34	42.08	38.9	37.91	35.87	36.59	32.98	25.12	23.87
6	59.96	52.67	45.87	42.24	41.29	39.5	40.31	39.64	30.19	28.92
7	70.1	68.97	52.98	50.07	50.89	47.89	50.1	46.82	35.79	32.96
8	83.96	80.14	60.1	58.98	55.12	52.15	58.26	55.97	40.16	39.71
9	88.98	86.43	66.78	63.45	63.91	61.2	62.64	59.87	46.76	42.65
10	92.6	91.23	72.12	69.08	71.18	69.53	69.63	67.5	50.13	48.71

*Mathematical modeling*

Correct determination of the release mechanism depends greatly on the selection and application of a suitable model to the release data. Model fitting of 10 h reveals that all the batches follow the matrix or Higuchi and Korsmeyer-Peppas model. The *R-*values in the case of all batches were higher for the Korsmeyer-Peppas model. The values of *n *suggest that all formulations followed Super case II transport release mechanism from the nanoparticles. The *R-*values of model fitting data for 10 h show that Simvastatin release followed the zero-order and matrix/Higuchi model.

**Table 6:** Kinetic values obtained from *in-vitro* release profile of nanoparticles (Zero order and First order).

**Formulation**	**Zero order plot**			**First order plot**	
	**Regression coefficient (r)**			**Regression coefficient** **(r)**	
	**Plain nanoparticles**		**Coated nanoparticles**	**Plain nanoparticles**	**Coated nanoparticles**
F1	0.992	0.993		0.917	0.921
F2	0.993	0.995		0.982	0.978
F3	0.993	0.993		0.969	0.965
F4	0.995	0.995		0.975	0.987
F5	0.998	0.993		0.990	0.986

**Table 7:** Kinetic values obtained from *in-vitro *release profile of nanoparticles (higuchi, korsmeyer peppa.s models).

**Formulation**	**Higuchi's**				**Korsmeyer peppa's**			
	**Plain nanoparticles**		**Coated nanoparticles**		**Plain nanoparticles**		**Coated nanoparticles**	
	**Slope** **(n)**	**RegressionCoefficient** **(r)**	**Slope** **(n)**	**Regression Coefficient** **(r)**	**Slope** **(n)**	**Regression Coefficient** **(r)**	**Slope** **(n)**	**Regression Coefficient** **(r)**
F1	32.28	0.921	30.97	0.945	0.898	0.985	0.897	0.984
F2	24.55	0.931	27.63	0.936	0.924	0.986	0.962	0.988
F3	26.72	0.946	26.98	0.956	0.987	0.983	0.978	0.980
F4	23.81	0.908	25.48	0.938	0.921	0.986	0.989	0.985
F5	19.22	0.943	17.4	0.943	0.921	0.997	0.923	0.978

*Receptor - ligand binding study*

From the study, it was found that the amount of dug release from the formulation F3 after 10 h was only 5.67%, prior to that the release was 42.09%.So, the remaining 36.42% drug binds with receptor present in hepatocytes.

## Conclusions

It can be concluded from the above studies that it is possible to prepare Simvastatin nanoparticles using bovine serum albumin as a macromolecular material with controlled release up to 10 h. The mathematical model fitting of the release data showed that the formulations followed case II transport mechanisms. 

The accumulation of galactose-coated albumin nanoparticles in liver due to their preferential macrophage uptake by RES organs. After administration, nanoparticles are selectively taken up by the macrophage rich organs by receptor-mediated endocytosis due to the presence of asialoglycoprotein receptor on the cell surface. After reaching inside the cell, these nanoparticles are degraded by lysosomes and entrapped Simvastatin is release, which is a potent competitive inhibitor of 3-hydroxyglutaryl-CoA reductase the enzyme that catalyzes the rate-limiting step of cholesterol biosynthesis. 
